# Value of Academic and Community Partnerships in Developing Age-Friendly Healthcare Systems Across Two Counties

**DOI:** 10.1093/geroni/igaa057.2960

**Published:** 2020-12-16

**Authors:** Roopali Gupta, Phillip Greiner, Jennifer Reichstadt, Kellie Scott

**Affiliations:** 1 University of California, San Diego, San Diego, California, United States; 2 San Diego State University, San Diego, California, United States; 3 University of California San Diego, San Diego, California, United States; 4 University of California San diego, San Diego, California, United States

## Abstract

The San Diego Imperial Geriatric Education Center is comprised of a robust partnership between two academic institutions, two County Area Agencies on Aging, three Federally Qualified Health Centers (FQHCs), three Programs of All-Inclusive Care for the Elderly (PACE), and several local community organizations providing older adult services across two large, diverse counties. Guiding the implementation of Age-Friendly and Dementia-Friendly Healthcare at our partner FQHC and PACE sites, and a large academic institution, has allowed us to review the similarities and unique qualities of each organization. We identified a common theme that providers would benefit from improved awareness and access to community resources (e.g., cognitive assessment and other ADRD (Alzheimer’s Disease and Related Dementias) support services), regardless of the specific health care system. The value of academic and community partnerships and the development of an infrastructure for information sharing and linking resources will be highlighted during this symposium presentation.

